# Toxicity, Biocompatibility, pH-Responsiveness and Methotrexate Release from PVA/Hyaluronic Acid Cryogels for Psoriasis Therapy

**DOI:** 10.3390/polym9040123

**Published:** 2017-03-27

**Authors:** Cătălina Natalia Cheaburu Yilmaz, Daniela Pamfil, Cornelia Vasile, Nela Bibire, Raoul-Vasile Lupuşoru, Carmen-Lăcrămioara Zamfir, Cătălina Elena Lupușoru

**Affiliations:** 1 Department of Physical Chemistry of Polymers, “Petru Poni” Institute of Macromolecular Chemistry of the Romanian Academy, Iași 700487, Romania; duncaty@gmail.com (C.N.C.Y.); pamfil.daniela@icmpp.ro (D.P.); 2 Department of Pharmaceutical Technology, Faculty of Pharmacy, Ege University, Izmir 35100, Turkey; 3 Department of Analytical Chemistry, Faculty of Pharmacy, “Grigore T. Popa” University of Medicine and Pharmacy, Iaşi 700115, Romania; 4 Department of Pathophysiology, Faculty of Medicine, “Grigore T. Popa” University of Medicine and Pharmacy, Iaşi 700115, Romania; rvlupusoru@yahoo.com; 5 Department of Histology, Faculty of Medicine, “Grigore T. Popa” University of Medicine and Pharmacy, 16 Universităţii Street, Iaşi 700115, Romania; zamfircia@yahoo.com; 6 Department of Pharmacology, Faculty of Medicine, “Grigore T. Popa” University of Medicine and Pharmacy, Iaşi 700115, Romania; celupusoru@yahoo.com

**Keywords:** methotrexate, cryogel, acute toxicity, biocompatibility, release, pH responsiveness

## Abstract

Poly(vinyl alcohol)/hyaluronic acid cryogels loaded with methotrexate were studied. The physical–chemical characterization of cryogels was performed by FT-IR spectroscopy, scanning electron microscopy, differential scanning calorimetry and dynamic mechanical thermal analysis. Acute toxicity and haematological parameters were determined by “in vivo” tests. The biocompatibility tests proved that the obtained cryogels showed significantly decreased toxicity and are biocompatible. The pH-responsiveness of the swelling behaviour and of the methotrexate release from the poly(vinyl alcohol)/hyaluronic acid (PVA/HA) cryogels were studied in a pH interval of 2–7.4. A significant change in properties was found at pH 5.5 specific for treatment of affected skin in psoriasis disease.

## 1. Introduction

Biodegradable and biocompatible hydrogels used in drug delivery are obtained by both physical and chemical crosslinking strategies. By optimizing synthesis conditions, the physical and chemical properties of hydrogels can be controlled. By their relative deformability they can conform to the shape of the surface to which they are applied, and their muco- or bioadhesive properties are advantageous when used with intent to immobilize them at the site of application [1]. Before use in disease therapy, the obtained hydrogels are loaded with drugs. Both quantity and homogeneity of drug loading depend on the morphology of hydrogels, swelling behaviour, and also on drug solubility. All these characteristics finally determine drug release. Therefore, the properties of each hydrogel should be tailored according to the desired drug delivery application.

According to O’Daly [2] and Irvington et al. [3] “psoriasis is a chronic inflammatory skin disease that affects 1% to 3% of the world population, with equal gender distribution” and it is appreciated that the incidence rates vary from 50 to 140 cases per 100,000 people per year [4]. Severe psoriasis is associated with a decrease in the life quality of and risk of mortality [5]. 

Active treatment for psoriasis using topical agents [6] is typically sufficient to manage mild or moderate psoriasis [7] when it affects less than 10% of the body surface area [8]. With increasing severity of disease, other therapies are necessary, which sometimes involve non-biological systemic agents such as methotrexate, cyclosporine and orally administrated retinoids [9].

Methotrexate (amethopterin, 4-amino-N10-methyl petroyl glutamic acid, MTX) is clinically used in formulations for therapy of several diseases such as acute lymphoblastic anaemia, choriocarcinoma, psoriasis, sarcoidosis, and trophoblastic tumours, etc. MTX inhibits epidermal cell proliferation [10] and has anti-inflammatory action at low doses [11]. However, the systemic administration of MTX leads to a large number of adverse effects (such as liver toxicity, and gastrointestinal side-effects, including nausea, vomiting, diarrhoea and stomatitis) which restrict its use. To overcome these limitations, its loading in matrices resulting from the combination of natural and synthetic polymers was developed as an important strategy, and also various formulations containing methotrexate have been developed. As methotrexate possesses two aromatic –NH_2_ and two aliphatic –COOH groups, Riebeseel et al. esterified carboxylic groups with hydroxyl group of polyethylene glycol (PEG) and developed drug delivery systems based on methotrexate PEG esters [12]. Pinto et al. [13] reported nanostructured lipid carriers loaded with MTX with good results for the in vitro skin permeation studies. Goodarzi et al. [14] reviewed the role of polysaccharides in the improvement of the cytotoxic drug delivery by increasing their water solubility and their safe use through a prolonged, targeted release [15].

Among natural and semi-synthetic polysaccharides, hyaluronic acid, chitosan and dextran are preferred in bioconjugation studies [16] as the therapeutic properties of the drugs can be combined with the biological properties of these polymers.

Hyaluronic acid (HA) is one of the most important topical carriers for the localized delivery of drugs to the skin and also as a drug delivery agent for ophthalmic, nasal, pulmonary, parenteral and topical routes of administration [17]. HA acts as mucoadhesive, retaining the drug at specific site of action/absorption. It also can modify the in vivo release/absorption rate of the therapeutic agent and it is able, when applied topically, to localize delivery of drug to the epidermis [18].

Poly(vinyl alcohol) (PVA) is a water soluble biodegradable synthetic polymer with good biocompatibility, and it can be physically cross-linked by the freeze-thaw method [19] to form hydrogels useful in pharmaceutical formulations [20].

HA–PVA hydrogels were prepared by Kim et al. [21] and Ramires and Milella [22] in the presence of a chemical crosslinker (glutaraldehyde) and 1-ethyl-(3-3-dimethylaminopropyl) carbodiimide hydrochloride (EDC) as a coupling agent. They obtained pH- and electro-responsive hydrogels with different compositions. The presence of glutaraldehyde residuals unremoved by washing procedures imparts toxicity to the materials.

Freeze-thawed PVA [23] and PVA/HA cryogels exhibited nontoxic behaviour and higher cell viability [24].

In a previous paper [25] the freeze-thawed PVA/HA hydrogels were prepared and tested as carriers for melatonin.

Loading of MTX within a hyaluronic acid-based matrix was done previously by using a peptide linker by Homma et al. [26], which was designed to be used in the treatment of osteoarthritis, localizing the drug at the synovial membrane.

Methotrexate-loaded freeze-thawed HA/PVA hydrogels were not still found to be reported. 

The aim of the present study is to prepare MTX-loaded freeze-thaw HA/PVA hydrogels and to characterize them in respect with the previous results on unloaded hydrogels and to determine their physical–chemical properties which assure a controlled release of the drug. The major outcome of the study is to obtain good protection of the drug and to reduce the reported side effects of MTX while it is carried and delivered to the affected site. This was done by analysing the acute toxicity and biocompatibility and by establishing the dependence of swelling and release behaviour on pH.

## 2. Materials and Methods

### 2.1. Materials

Hyaluronic acid sodium salt, from *Streptococcus equi*, was acquired from Sigma-Aldrich. Polyvinyl alcohol with 99% hydrolysis degree and a *M*_w_ of 89,000–98,000 as well as methotrexate, were purchased from Sigma-Aldrich (Buchs, Switzerland). All other chemicals and reagents used in this study were of analytical grade or higher, obtained commercially.

Hydrogels of PVA and HA prepared by method elaborated by Fahmy et al. [27] which was a modified as described in the procedure given in previous paper [25]. MTX was dissolved in the solution of HA with a concentration of 0.5% (*w*/*w*) in respect to polymeric matrix, and homogenized with vortex for 5 min and then added to the PVA solution drop-wisely. The volume ratio between the two solutions was PVA/HA 5:1 (*v*/*v*). The freeze-thaw procedure consists of freezing at −20 °C for 20 h and thawing for 4 h at room temperature. The formed gels were frozen and freeze-dried right after the freeze-thaw process. Applying the same procedure of consecutive freeze-thaw cycles resulted in MTX-loaded samples. Increasing or decreasing the freeze and thaw action time has a significant influence of the mechanical properties of the final gels. A list of samples and assigned codes are summarized in Table 1.

### 2.3. Investigation Methods

In this paper the freeze-thawed MTX loaded PVA/HA hydrogels underwent physical–chemical characterization and were analysed with respect to with toxicity and biocompatibility. Physical–chemical characterization of the PVA/HA hydrogels occurred using the following methods: FT-IR spectroscopy (ATR-FT-IR spectra were recorded using a Perkin Elmer Spectrum 100 Spectrometer (Shelton, CT, USA), through reflexion on a diamond crystal with an angle of 45 degrees, resolution 4 cm^−1^), differential scanning calorimetry (DSC) (Perkin Elmer DSC-8000, Shelton, CT, USA within a temperature range of 30 and 220 °C at a heat rate of 10 °C/min under 20 mL/min nitrogen flow), and dynamic mechanical–thermal analysis (DMTA) (Anton Paar MCR301 Rheometer, Physica MCR, Berlin, Germany); polymer membranes under half-wet state with rectangular shape of 40 mm × 12 mm dimensions at a constant frequency of 1 Hz from −50 to 220 °C temperature range) was performed according to the procedures and experimental details described in previous paper [25]. Here, only particular behaviour of MTX-loaded hydrogels is presented but sometimes reference to the previous results on unloaded hydrogels is made.

### 2.4. Drug Loading Evaluation

The loaded amount of methotrexate was evaluated by using HPLC method. An AGILENT 1200 Series, USA HPLC System (Agilent, New York, NY, USA) with vacuum Degasser, UV detector and ACE 5^®^ equipped with a column of Agilent Eclipse XDB-C18 with a length of 150 mm and a 4.6-mm internal diameter, particle size 5 µm was used. A degassed solution containing 90% (*V*/*V*) phosphate buffer pH 5.5 and 10% (*v*/*v*) acetonitrile was used as the mobile phase. The flow rate was set 1 mL/min and the UV detection was set at 302 nm, the characteristic wavelength of methotrexate. A modified HPLC method was used to determine amount of loaded methotrexate [28]. The method was validated in terms of specificity, stability of solution, accuracy and precision. The linearity range was 0.24–10 µg/mL and the correlation coefficient (*R*) was 0.989. The compounds were identified by comparing the retention times of the unknown peaks with the peaks of the reference standards. The retention time considered for methotrexate was found at 4.4 min. Weighed amounts of lyophilized hydrogels (0.027 g HA/PVA 2c-MTX and 0.03 g HA/PVA 3c-MTX) were placed in a vial and 10 mL of phosphate buffer pH 5.5 adjusted with NaOH 0.2 M were added. The systems were stirred overnight to ensure a good extraction of the drug from the polymeric matrix. Volumes of 1 mL were sampled for the HPLC characterization. The loading percent was calculated taking into account the initial amount of MTX loaded at the beginning of the experiment (0.4 mg) and it was found to be 66%.

### 2.5. Toxicity and Biocompatibility Tests

#### Ethics Statement for Experiments with Animals

Experimental protocols within the toxicity and biocompatibility studies were implemented according to the institutional recommendations of the “Gr. T. Popa” University of Medicine and Pharmacy of Iasi, Romania (official paper No. 15559/21.09.2010) Committee for Research and Ethical Issues, in rigorous accordance with international ethical regulations on laboratory animal work [29,30].

The laboratory animals were euthanized by exposure to ether vapour overdose in closed containers, as ether is one of the inhalant anaesthetics considered acceptable conditionally. The administration path for the in vivo studies was selected based on the characteristics related to each medium.

Prior to the biocompatibility tests, PVA/HA hydrogels were mashed and then suspended in saline solution with Tween 80.

For the in vivo experiments white Swiss mice were used (25–30 g). The mice were housed under standard laboratory conditions (relative humidity 55–65%, room temperature 23.0 ± 2.0 °C and 12 h light/dark cycle), with food and water ad libitum.

In order to determine the acute toxicity, the Lethal Dose LD50 was calculated, after intraperitoneal single dose administration in mice, using Karber arithmetic method [31]. Both the minimal dose that kills all the animals (LD100) and the maximal dose that fails to kill any animal were determined. Between these two limits, different doses were chosen, and administered in groups of four animals [32]. During the subsequent 14 days after immediate dosing, the animals were carefully observed for any behavioural changes, mortality and any change correlated with the manifestations of toxicity (lack of appetite, depression, immobility, respiratory distress). Mortality rates in each group were counted, and the LD50 was calculated, according to the Karber method by using Equation (1):

LD50 = LD100 − ∑(*a* × *b*)/*n* (1)

where *a*—the difference between two successive doses of the substance administered, *b*—the average of the number of death animals in two successive groups, and *n*—the number of animals in a group.

The level of substance toxicity was appreciated according to the toxicity scale proposed by Voicu in 1997 [33]. As already mentioned, at the end of the observation period the animals were euthanized with an overdose of ethyl ether.

The biocompatibility properties of doses representing 1/20 from LD50 of the substances were studied in two situations. Firstly, the effects on the blood and biochemical constants were assessed, 14 days after intraperitoneal unique administration of the substances. The experiment was carried out on mice distributed in four groups of six animals as listed within Table 2, column 2. Each animal was treated intra-peritoneally, on the first day of the experiment.

The effect on the blood and biochemical parameters were determined after unique topical or intra-peritoneal administration for 7 or 14 days, respectively, of the loaded and unloaded gel formulations. The experiment was carried out on mice distributed in another four groups of six animals each treated topically with two drops of the formulations behind the left ear of the animal, on the first day of the experiment. The last column of the Table 2 summarizes the group samples for the topical administration of the gel formulations.

On the last day of both of the experiments, the blood was collected and with the automated haematological analyser (SySmex XT 1800 from SYSMEX Europe GmbH, Norderstedt, Germany) the following parameters were determined: white blood cell count and leukocyte formula, red blood cell count, platelet count, packed cell volume and haemoglobin levels. With the automated biochemical analyser RX Imola (from RANDOX laboratories, Crumlin, UK) the following parameters were determined in serum: aspartate aminotransferase, alanine aminotransferase, urea, creatinine, uric acid, total cholesterol and total proteins. After the experiments all animals were euthanized. After animal euthanasia, tissue fragments were collected for histological examination. The organ fragments were fixed in 10% formalin solution and prepared for light microscopic examination and stained with hematoxylin–eosin (HE).

### 2.6. Swelling Tests

Swelling ability of the matrices was investigated by means of swelling tests by direct immersion in buffered solutions (PBS) of various pHs as 2, 4, 5.5 and 7.4 at a temperature of 37 ± 0.5 °C, simulating physiological conditions. The analysed hydrogels were removed from the solution at pre-determined intervals, the excess solution on the surface was removed with a soft tissue, weighed and then carefully placed back into the vessel with swelling solution as soon as possible for the next determination.

The following equation was used to evaluate the swelling degree (*Q*):

(2) $$Q(\%)=\frac{\left(W_{t}-W_{d}\right)}{W_{d}}\times 100$$

where *W*_t_ is the weight of the swollen samples at time *t* and *W*_d_ is the weight of the dry sample.

The swelling kinetic parameters have been evaluated by means of equations from Peppas et al. [34,35,36]:

(3) $$\frac{W_{t}}{W_{\mathrm{eq}}}=k_{\mathrm{sw}}\;t^{\mathrm{nsw}}$$

where *W*_t_ and *W*_eq_ are the amounts of solution absorbed by polymeric hydrogels at time *t* and at equilibrium, respectively; *k*_sw_ is the swelling specific rate constant of the studied sample and *n*_sw_ is the exponent in diffusion law expression whose values define the mechanism of the solvent transport. Equation (3) is valid for a swelling degree less than 60% where the plot of ln*W*_t_/*W*_eq_ versus ln*t* gives a straight line.

### 2.7. In Vitro Release of MTX from Cryogels

The in vitro release of the methotrexate at various pH values of 2, 4, 5.5 and 7.4 were performed by using a standard dissolution test [37] carried out in conditions which mimic the physiological environment at a temperature of 37 ± 0.5 °C. The concentration of the drug was evaluated using a predetermined calibration curve for MTX recorded using an HP 8450A UV-visible spectrophotometer (Agilent, San Diego, CA, USA)—specific maximum absorption wavelength of 303 nm.

As in the case of swelling kinetics for the drug release kinetics, Korsmeyer–Peppas semi-empirical Equation (4) was applied for the initial release stages (~60% fractional release) [36]:

(4) $$\frac{M_{t}}{M_{\infty }}=k_{r\;}t^{n_{r}}$$

where $M_{t}/M_{\infty }$ is the fractional drug released, *M*_t_ and $M_{\infty }$ are the cumulative drug released amounts at time *t* and at equilibrium, respectively (or experimental maximum released amount taken at the plateau of the release curves), *k*_r_ is rate constant dependent on the characteristics of the drug loaded system and *n*_r_ is the diffusional exponent which defines the type of the release mechanism. For example, a value of *n*_r_ ~ 0.5 is characteristic of the Fickian diffusion mechanism of the drug from the cryogel; the values in interval 0.5 < *n*_r_ < 1 are specific to an anomalous or non-Fickian behaviour. A case II of transport mechanism occurs when *n*_r_ = 1, which means zero-order kinetics, while a special case II of transport mechanism is indicated by values *n*_r_ > 1 [35]. The MTX release profiles are plotted as the cumulative percentage of drug released versus time.

### 2.8. Statistical Analysis

The triplicate tests were performed and the obtained results are expressed as means ± SD (standard deviation) and significance was analysed using T-student test in Microsoft Excel for Windows. The StatView statistical software package (Apple Macintosh, BrainPower Inc., Cary, CA, USA) was used for data analysis. ANOVA and Fisher’s post hoc test consisting of 3 (groups) × 3 (time sample points) repeated measures of experimental results were analysed. The criterion for significance was *p* < 0.05.

## 3. Results and Discussion

### 3.1. Physical-Chemical Characterization

Methotrexate-loaded hydrogels were evaluated by comparison with the unloaded hydrogels by the above-mentioned methods. 

**Figure 1:**
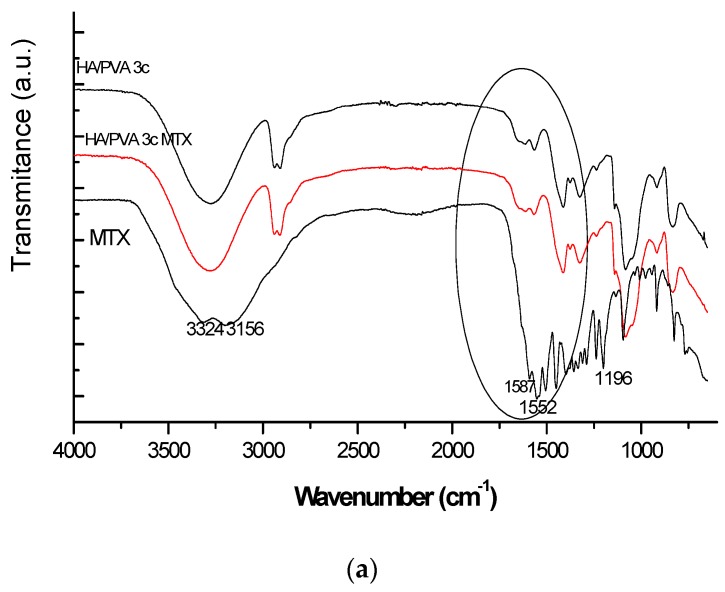

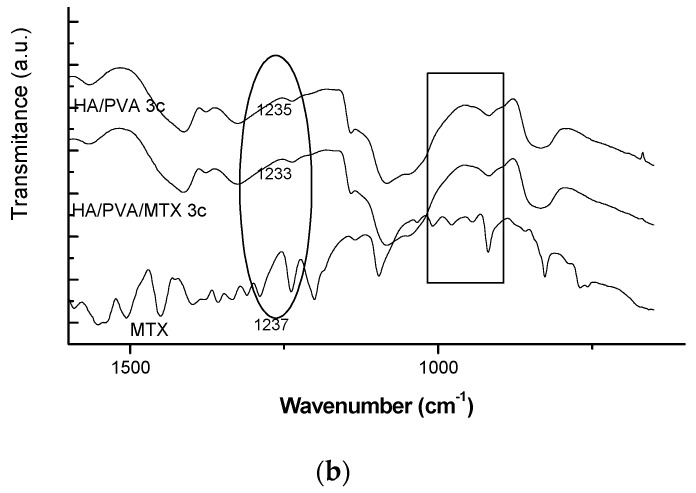
ATR-FT-IR spectra of HA/PVA unloaded and MTX-loaded cryogels (**a**) for the entire spectral range (**b**) for spectral range between 1800 and 500 cm^−1^.

In the MTX spectrum the following characteristic absorption bands are evidenced: a broad signal at 3350 cm^−1^ (assigned to O–H stretching from carboxyl groups superposed with the O–H stretching from crystallization water) and a band corresponding to primary amine N–H stretching centred at 3150 cm^−1^—Figure 1a. 

The vibration bands within the spectral region 1670–1600 cm^−1^ can be attributed to the C=O stretching vibration from carboxylic group and to that of C=O stretching from the amidic group. In the 1550–1500 cm^−1^ spectral range bands assigned to N–H bending from amidic group which partly overlapped with the aromatic –C=C stretching are found—Figure 1b. Another prominent band was determined within 1400–1200 cm^−1^ corresponding to –C–O stretching from carboxylic group. As the amount of loaded MTX was theoretically ~ 0.5% with respect to matrix, significant differences are not clearly visible excepting some of band shifts marking H-bonds from 3274 to 3257 cm^−1^ and from 1414 to 1409 cm^−1^ between the active substance and the polymeric matrix. 

**Figure 2:**
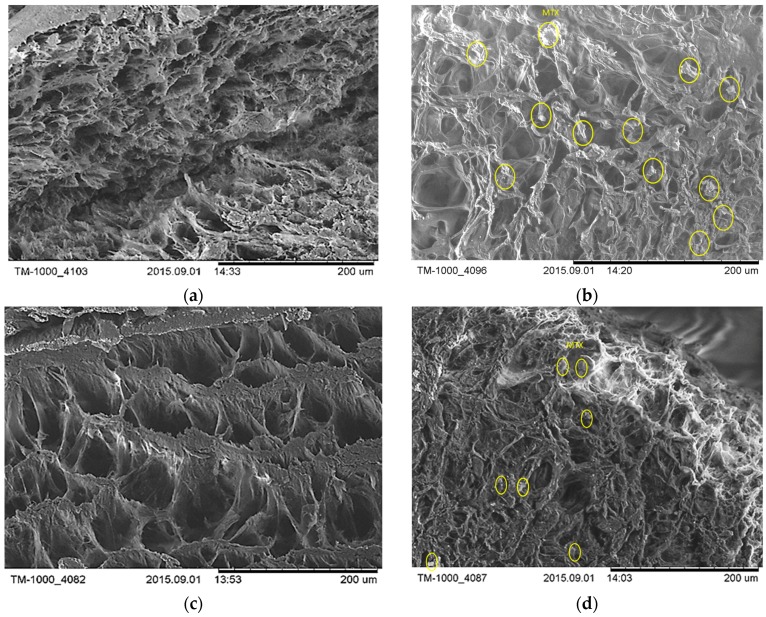
SEM images of (**a**) HA/PVA 2c; (**b**) HA/PVA/MTX 2c; (**c**) HA/PVA 3c; (**d**) HAPVA/MTX 3c.

SEM images of the unloaded and loaded cryogels are shown in Figure 2.

SEM images confirmed that the methotrexate was dispersed within the polymeric matrices being observed as uniformly dispersed stick-shaped particles. The size of pores for unloaded HA/PVA cryogels varied from 1.5 to 7 μm while loaded matrices with MTX had diameter dimensions in the range of 1.5 to 4 μm for HA/PVA/MTX 2c and 2.3–4.4 μm for HAPVA/MTX 3c. By loading hydrogels with MTX, the pore sizes are slightly decreased, indicating a good compatibility between the HA/PVA matrices and active substance. The determined transition temperatures for the HA/PVA hydrogels, read from the dependencies of dynamic moduli and damping factor on the temperature, are listed in Table 3.

*T*_g_ of PVA found around 65 °C [38] is strongly affected by the water content as demonstrated for some hydrophilic polymers [39]. In the case of freeze-thawed hydrogels, water induced a plasticizing effect on the polymer, decreasing *T*_g_ up to 39 °C.

The low-temperature peaks were evidenced indicating the presence of freezable bound water within the polymeric network which show transition temperatures at very low values i.e., at −19 °C for HA/PVA3c and −4 °C for HA/PVA2c in accordance with results reported by other authors [38,40,41]. Transition temperatures at higher temperature were assigned to the relaxation mechanism of the glycosidic bonds in HA and structural rearrangements due to the loss of the residual water [42]. The melting temperature of PVA at 167–177 °C increases with number of freeze-thaw cycles applied and decreases with MTX loading.

As can be observed, the loading of the drug inside the HA/PVA hydrogel caused a small decrease of transition temperatures in both cases as a result of good compatibility of methotrexate within the network of HA/PVA.

**Figure 3:**
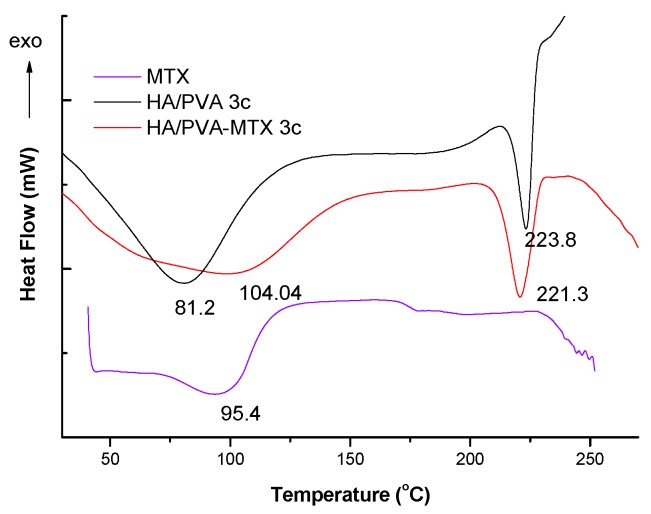
Differential scanning calorimetry (DSC) curves of the MTX and of unloaded and MTX-loaded PVA/HA cryogels.

In the DSC curves, two endothermic processes are present as shown in Figure 3. The first was assigned to water loss at 81.2 °C for the unloaded PVA/HA 3c cryogel, shifting to a higher temperature of 104 °C in the case of the MTX-loaded cryogel, probably because of stronger interactions with water. The melting temperature of PVA in the MTX-loaded cryogel is decreased with respect to the unloaded one because of the depression of melting temperature caused by the presence of the uncrystallisable components.

### 3.2. Toxicity and Biocompatibility Studies

The acute toxicity of a high dose was tested according to OECD guidelines [43,44]. During the entire period of examination, animals whose unloaded and MTX-loaded cryogels were administered did not show any signs of peritonitis, lethargy, muscle loss, dehydration or anorexia, symptoms which are commonly associated with toxicity of the studied samples [45]. In the case of unloaded HA/PVA hydrogel, LD100 after i.p. administration was 4000 mg/Kg body weight (kbw), in mice. The value of the LD50 for hydrogel administered intra-peritoneally as a single dose in mice is 2937.5 mg/kbw; they survived 14 days after administration, with results showing their non-toxicity. Table 4 summarize the results obtained for DL50 values for all the formulations and the statistics related to the survival rate of mice.

As already mentioned, MTX is known for its toxicity to the liver if it is directly administered orally or via injection. The hepatic, pulmonary, renal and bone marrow abnormalities manifest as major toxic effects, which must carefully monitored, as well as the minor toxic effects, such as stomatitis, malaise, nausea, diarrhoea, headaches and mild alopecia [10,11,45]. The HA/PVA cryogels are not cytotoxics—Table 4—and loading into PVA/HA hydrogel reduces MTX toxicity. 

The LD100 value obtained in the case of i.p. administration at mice with HA/PVA2c-MTX (D2Y) was 2000 mg/kbw, in mice. The value of the LD50 for HA/PVA2c-MTX administered i.p. at mice as a single dose in mice was 906.25 mg/kbw. As no more deaths were recorded in the following hours, the LD50 in mice has the same value after 24 h, 48 h, 72 h and 14 days.

In the case of matrix consisting of HA/PVA 3c-MTX, LD100 after i.p. administration of hydrogel at mice was 2000 mg/kbw. Furthermore, the DL50 was calculated as being 1250 mg/kbw. As no more deaths were recorded in the following hours, the LD50 in mice had the same values after 24 h, 48 h, 72 h and 14 days. The value of DL50 of 1250 mg/kbw included the hydrogels within the class of materials with a slightly toxic action [33] and permitted further research on biocompatibility.

The in vivo biocompatibility of the HA/PVA cryogels was examined a period of 14 consecutive days after intra-peritoneal injection of gel suspensions at mice and it has been assessed by the determination of the haematological and immune system parameters comparatively with a control group of mice, which received just physiological serum with few drops of Tween 80 (Table 2).

There were no statistical significant modifications of the haematological and biochemical constants assessed from the blood of the animal groups treated with hydrogels, compared to the animals in the control group (*p* > 0.05) for mice after i.p. unique treatment (14 days after the experiment) nor for mice after topical unique treatment (7 days after the investigation)—Table 5. 

Clinical chemistry and haematology data are of great importance to determine the effects induced on the mice body by the tested hydrogels. The haematological parameters values, namely of white blood cells (WBC), red blood cells (RBC), platelets (PLT), haemoglobin level (HGB) concentration, and haematocrit level showed no significant variations between mice groups treated with HA/PVA hydrogels comparatively with those of the control mice group, all of them being in the range of normal limits reported for healthy mice—Table 5 [43]. 

No significant pathological changes in the tissue samples were found from animals treated with hydrogels in doses representing 1/20 from LD50, compared to the control group. 

### 3.3. Histopathologic Examination

**Figure 4:**
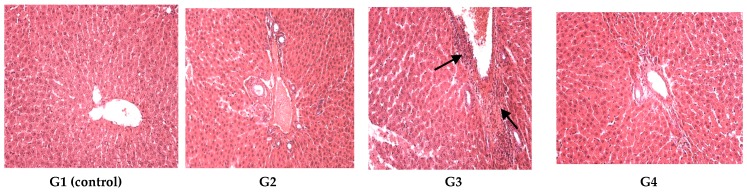
Liver reactivity: in G1 (control), G2, G4 normal hepatic morphology was maintained; G3 revealed a dilated congestive central vein and inflammatory areas. The arrows in G3 indicate some alterations.

The liver did not present signs of alterations in G1 (control), G2, G4; the one-cell-thick hepatic cords were normally distributed and hepatic vasculature had a normal configuration. G3 presented hepatic alterations following D2Y administration: a large distended portal vein, with perivascular disseminated inflammatory areas indicated by arrows in Figure 4. 

**Figure 5:**
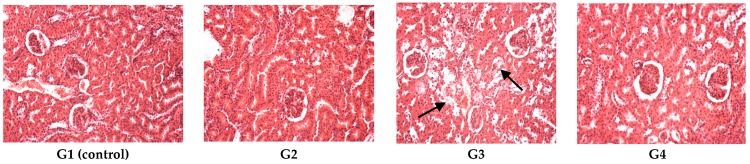
Kidney histopathologic exam revealed a normal renal structure for G1 (control), G2, G4, G3 revealed moderate grades of tubular epithelial degeneration. Hematoxylin–eosin (HE) stain, ob. ×20. The arrows in G3 indicate some alterations.

The kidney had a normal configuration in G1 (control), G2, G4, while in G3, epithelial degeneration was observed in the renal tubules; tubular alterations occurred in most of the proximal convoluted tubules and to a lesser extent in the distal tubules (indicated by arrows in Figure 5). The glomeruli and the renal blood vessels remained intact (Figure 5). 

**Figure 6:**
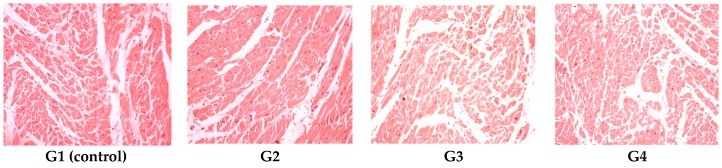
Cardiac histopathologic exam revealed a normal histologic aspect of the heart for G1 (control), G2, G3 and G4. HE stain, ob. ×20.

The heart was normally configured in all the experimental groups (Figure 6).

**Figure 7:**
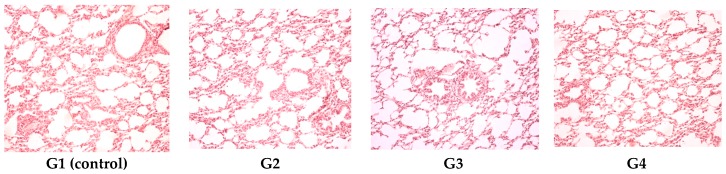
Pulmonary histopathologic exam revealed a normal configuration of the lungs for G1 (control), G2, G3 and G4. HE stain, ob. ×20.

The lungs did not present alterations in any of the investigated experimental groups (Figure 7).

**Figure 8:**
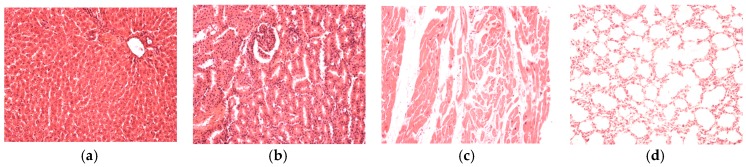
Normal tissue morphology after topical administration of D2Y (G3T): (**a**) liver; (**b**) kidney; (**c**) heart; (**d**) lung. HE stain, ob. ×20.

We did not find significant pathological changes in the tissue samples from animals topically treated with hydrogels (G2T, G3T, G4T,) in doses representing 1/20 from LD50, compared to the control group (G1T). Figure 8 shows samples from G3T.

### 3.4. pH-Dependence of the Swelling and Drug Release Behaviour

Materials which shrink/swell in response small variations in pH, temperature, or ionic strength of the environment are of great interest in many applications, especially in medical and pharmaceutical ones. Among these materials, stimuli-responsive hydrogels have received special interest in medical applications because of their hydrophilicity and biocompatibility and soft physical properties associated with living tissues as well as very good control of these in response to external changes which allow an effective treatment of various diseases [46,47,48,49].

As PVA/HA cryogels are intended to be applied in drug delivery, both swelling and release behaviour under external stimuli should be investigated.

#### 3.4.1. Swelling Behaviour

To understand the swelling and release behaviour of the PVA/HA/MTX hydrogels, both matrix and drug characteristics should be considered. 

Maleki et al. [50] and Burke et al. [51] studies demonstrated the pH-dependence of the HA behaviour in solution [50,51]. As the physical–chemical characterization of the cryogels was presented above, some data on methotrexate mainly in respect with structure and solubility are summarized, while other data have already been mentioned. 

Methotrexate is a chemotherapeutic drug that is structurally similar to folic acid. 

**Figure 9:**
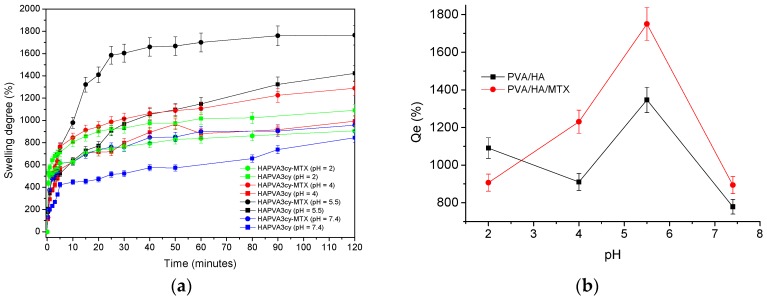
Swelling profiles at different pHs values of the unloaded and MTX-loaded HA/PVA based matrices (**a**) and dependence of the *Q*e on pH (**b**).

MTX is used in the treatment of a variety of illnesses including cancer, rheumatoid arthritis, systemic lupus erythematosus, and psoriasis. It is delivered intravenously, intramuscularly, orally and intrathecally. MTX toxicity develops due to increased patient susceptibility during treatment, excessive parenteral or intrathecal administration, therapeutic errors by patients, self-administration to induce abortion, or intentional oral overdoses. Toxic effects may occur hours to days to weeks after MTX administration or overdose [52]. MTX is slightly soluble in dilute hydrochloric acid, but is soluble in dilute solutions of alkali. A 20- and 12-fold increased solubility was found when pH increased from 5 to 7. Aqueous solutions can be prepared by dissolving in aqueous buffers. The solubility in PBS 7.2 is ~1 mg/mL. It is not recommended to store solution more than one day [53]. Several p*K_a_* values are given in literature for MTX as 4.70 5.6, 4.8, and 3.8 (carboxylic acid) [54,55]. All these characteristics of the HA and MTX will give a pH responsiveness of the MTX-loaded PVA/HA cryogels. Swelling profiles of the unloaded and MTX-loaded HA/PVA cryogels in the media of various pH are presented in Figure 9a.

**Figure 10:**
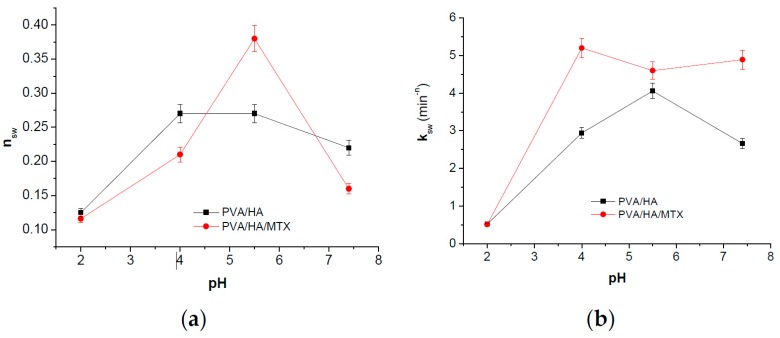
Dependence of the *n*_sw_ (**a**) and *k*_sw_ (**b**) on pH.

For the loaded hydrogels with MTX, values are higher (1412.6% for HAPVA2cy-MTX and 1749.9% for HAPVA3cy-MTX)—Figure 10b and Table 6—than those for unloaded cryogels due to the additional contribution of MTX with OH, NH_2_ and COOH groups to the hydrogel network. The swollen HA/PVA cryogels have a soft consistency, which is required for the topical application of methotrexate. A dependence on pH with respect to the swelling behaviour, mainly due to the HA presence [56], is obtained—Figure 9b. A maximum of *Q*esw appears at pH 5.5 in accordance with other literature data [56,57] The values of swelling kinetic parameters are also dependent on pH values as this is clearly observed from the data in Figure 10 and Table 6.

As it could be observed, all *n*_sw_ values are below 0.5, which led to the conclusion that an anomalous mechanism of swelling occurred. This case is called a “less Fickian” behavior as already mentioned above but is also the case for swelling with other hydrogels [58,59,60]. 

Incorporation of drug does not change *n*_sw_ values but the specific rate constant is a little increased. The highest values of *n*_sw_ and *k*_sw_ are found for pH 5.5 corresponding to a critical value of pH where some changes in swelling mechanism may occur. Similar data were obtained for systems resulted from two cycles of freeze-thaw. 

#### 3.4.2. In Vitro Release Studies

As determined from HPLC measurements, a total amount of 0.03 mg/mL of MTX was found. Drug loading tests performed prior to in vitro release studies revealed that the matrices are able to uptake approximately 60% (*w*/*w*) from the initially loaded drug. 

**Figure 11:**
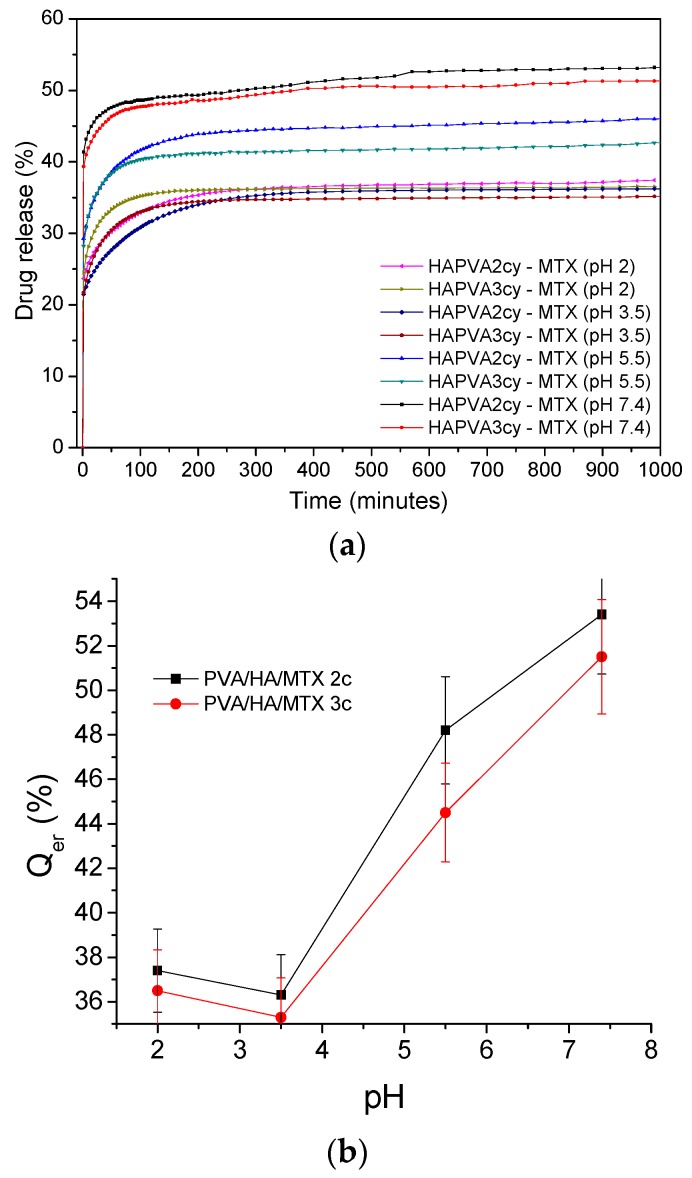
Kinetic release profiles of MTX from HA/PVA-based cryogels (**a**) and variation of release amount at equilibrium (*Q*_er_) with pH (**b**).

The release profiles of MTX from HA/PVA hydrogels as the variation in time of the MTX percent amount released in media of various pH are shown in Figure 11. When the in vitro release was measured by using release media with different pH, different profiles were observed—Figure 11a and Table 7. In the first 50 min, the release profiles showed a slight burst release, and the amount released represented approximately 25% and 40% (*w*/*w*) from the loaded amount of MTX. In the following interval, the drug was released slowly and a total amount of drug released was of 35–53.4% was released over 16 h. The *Q*_er_ values are slightly higher for cryogels prepared using two freeze-thaw cycles than those corresponding to three cycles. These differences are due to the smaller pores sizes which lead to the increase the time to reach equilibrium and greater amount of drug will be released in the media of various pH.

The release amount at equilibrium (*Q*_er_) depends on pH of the medium showing a sudden increase when pH > 4—Figure 11b. Vaidyanathan et al. [61] found that both drug solubility and steady-state penetration rate were significantly higher at pH 5.29 than at pH 4. Also, the increase of the vehicle pH to 6.34 the drug solubility is improved as well as steady-state rate of penetration while the percent amount penetrated decreased. Both *t*_eqr_ and *t*_1/2r_ increased with increasing pH value and the increase is very significant for pH > 4—Table 7.

**Figure 12:**
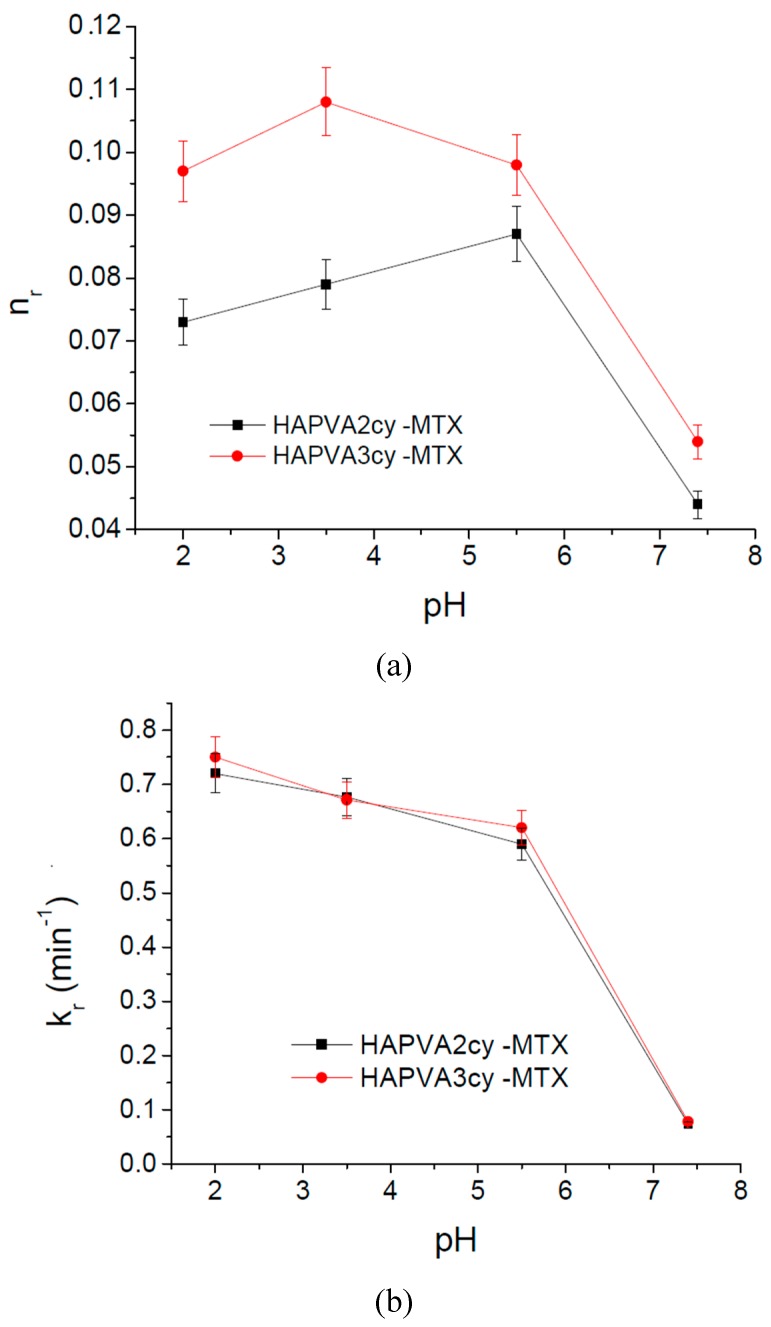
pH effect of on release parameters of MTX from PVA/HA matrices (**a**) diffusion exponent *n*_r_ and on the (**b**) kinetic constant *k*_r_.

These results were supported also by the kinetic parameters calculated based on the release profile and applying Korsmeyer–Peppas Equation (4) [36] as shown in Table 7 and Figure 12. 

The *n*_r_ values ranging between 0.04 and 0.11 indicate that the release mechanism of MTX from polymeric cryogels follows a “less-Fickian” diffusion. The small values recorded by *n*_r_ parameter in the basic medium (pH 7.4) are correlated with a slow drug release profile and are caused by the rate of polymer relaxation which is much greater than the rate of drug diffusion, and also with change in drug solubility. Both diffusion exponent *n*_r_ and kinetic constant *k*_r_ values show a sudden decrease at pH 5.5—Figure 12a,b. That means a change in the release mechanism which becomes more “less Fickian”. It can be concluded that both swelling and release mechanism of MTX from PVA/HA cryogels is changed around a critical pH of about 5.5, these systems showing pH-responsiveness. As pH value of 5.5 is characteristic for affected skin disease, it can be supposed that the MTX release at this pH favors psoriasis treatment as toxicity is also reduced by using PVA/HA hydrogels as carriers.

The variation of the kinetic parameter values is asociated with the p*K*_a_ values of the components and their interactions by functional groups, as was demonstrated for other pH-responsive systems [62,63,64,65,66], and in this case those of MTX and HA.

A decrease of the *k*_r_ values is associated with a slow drug release. It can be concluded that these matrices can be used for the type of systems to deliver low doses of drug i.e., topical applications.

## 4. Conclusions

The methotrexate-loaded hyaluronic acid and polyvinyl alcohol hydrogels were prepared by freeze-thaw techniques. By varying the freeze-thaw cycles two matrices were obtained which were differentiated by means of their physico-chemical properties and morphology. The differences found in physico-chemical properties of the matrices were not consistent, but were great enough to be able to modulate the amount of drug delivered. The acute toxicity studies proved that the matrices are non-toxic and relatively toxicologically safe when administered i.p. or topically in mice. The hydrogels determined similar haematological parameter modifications and biochemical responses with saline solution, after intra-peritoneal or topical administration in doses representing 1/20 from LD50, in mice. Using these doses, no major hystopathological variations compared to the control group were found. The PVA/HA/MTX cryogels showed pH-responsiveness both for swelling and release which is determined both by HA component of matrix and by the drug solubility. Due to the good in vivo biocompatibility of these hydrogels after systemic or topical administration in laboratory animals and the pH-responsive swelling and releasing abilities, these systems can be developed as topical formulations for psoriasis therapy.

## Figures and Tables

**Table 1:** Freeze-thawed poly(vinyl alcohol)/hyaluronic acid (PVA/HA) cryogels studied.

PVA/HA cryogels	Code
HA/PVA—2 cycles from −20 to 25 °C	HA/PVA 2c
HA/PVA loaded with methotrexate—2 cycles from −20 to 25 °C	HA/PVA/MTX 2c
HA/PVA—3 cycles from −20 to 25 °C	HA/PVA 3c
HA/PVA loaded with methotrexate—3 cycles from −20 to 25 °C	HA/PVA/MTX 3c

**Table 2:** Samples and assigned codes used for biocompatibility studies.

Sample	Code	Weight (mg/kbw) *	Code
Saline solution and Tween 80-reference	G1	0.1 mL/10 g body mouse weight	Control—G1T
HA/PVA 3c	G2	146.875 mg/kbw	G2T
HA/PVA/MTX 2c	G3	45.31 mg/kbw	G3T
HA/PVA/MTX 3c	G4	62.5 mg/kbw	G4T

* Kbw—Kg/body weight.

**Table 3:** Transition temperatures determined from dynamic mechanical–thermal analysis (DMTA) curves.

Sample	Transition temperatures (°C)								
	From *G*′ curve	From *G*″ curve		From tanδ curve					
HAPVA2cy	8	−16s	167	-	16	65s	104	154	
HAPVA3cy	15	−19	177	−16	18	39	108	139	178
HAPVA2cy-MTX	11	−17	176	−16	17	41	104	120	174
HAPVA3cy-MTX	15	−12 s	164	−12s	18		106.5	122	177

s—shoulder.

**Table 4:** Calculation procedure of LD50 using Equation (1) in mice using Karber arithmetic method, 24 h after intra-peritoneal administration.

Parameters in Equation (1)	HA/PVA 2c				HA/PVA2c-MTX (D2Y)				HA/PVA23-MTX (D3WY)			
Doses (mg/kbw)	4000	3000	2500	2000	1500	1000	500	250	2000	1500	1000	500
Number. of mice/group	4	4	4	4	4	4	4	4	4	4	4	4
Deaths	4	3	0	4	3	3	1	0	4	3	1	0
*A*		1000	500		500	500	500	250		500	500	500
*B*		3.5	1.5		3.5	3	2	0.5	3.5	2	0.5	3.5
*a* × *b*		3500	750		1750	1500	1000	125		1750	1000	250
LD50, mg/kbw	2937.5				906.25				1250			

**Table 5:** Values of the haematological and biochemical parameters in mice treated i.p. with hydrogels single dose treatment at 14 and 7 (GT columns) days after the experiment.

Group	G1	G1T	G2	G2T	G3	G3T	G4	G4T
n	6	6	6	6	6	6	6	6
WBC (K/μL)	5.60 ± 0.11	5.50 ± 0.03	5.56 ± 0.13	5.60 ± 0.06	5.65 ± 0.10	5.61 ± 0.12	5.62 ± 0.15	5.55 ± 0.09
N (%)	26.93 ± 0.82	26.78 ± 1.08	27.02 ± 0.61	27.05 ± 0.44	27.20 ± 0.75	26.03 ± 1.82	26.54 ± 1.56	26.20 ± 1.71
L (%)	64.20 ± 0.88	65.48 ± 1.32	65.23 ± 1.14	63.12 ± 1.41	64.96 ± 1.10	64.81 ± 1.48	64.41 ± 1.20	66.81 ± 1.54
M (%)	6.13 ± 0.51	6.21 ± 0.92	6.48 ± 0.21	6.46 ± 0.56	5.96 ± 1.64	6.29 ± 0.17	6.28 ± 0.71	6.51 ± 0.49
E (%)	0.72 ± 0.47	0.89 ± 0.52	0.71 ± 0.51	0.86 ± 0.26	0.91 ± 0.23	0.72 ± 0.33	0.94 ± 0.14	0.87 ± 0.26
B (%)	0.85 ± 0.35	0.93 ± 0.42	0.72 ± 0.25	0.83 ± 0.25	0.96 ± 0.43	0.86 ± 0.41	0.88 ± 0.36	0.91 ± 0.31
RBC (mil/μL)	8.66 ± 0.06	8.52 ± 0.09	8.23 ± 0.25	7.98 ± 0.82	7.92 ± 0.68	8.31 ± 0.26	8.19 ± 0.18	8.69 ± 0.08
Hb (g/dL)	13.10 ± 0.09	13.40 ± 0.17	12.60 ± 0.37	12.80 ± 0.87	13.00 ± 0.15	13.90 ± 0.89	12.60 ± 0.21	13.80 ± 0.76
PCV (%)	41.60 ± 0.06	43.00 ± 0.84	40.40 ± 0.15	41.00 ± 0.28	41.60 ± 0.21	43.30 ± 0.71	40.00 ± 0.36	44.90 ± 1.21
PLT (K/μL)	828 ± 19	955 ± 39	953 ± 30	969 ± 54	828 ± 11	964 ± 47	922 ± 45	888 ± 31
ASAT (U/L)	136 ± 28	142 ± 27	140 ± 21	138 ± 19	135 ± 13	141 ± 18	138 ± 21	140 ± 31
ALAT (U/L)	64 ± 3	64 ± 8	67 ± 2	74 ± 2	68 ± 5	72 ± 5	69 ± 4	64 ± 7
urea (mg/dL)	47 ± 6	50 ± 4	53 ± 4	49 ± 7	50 ± 5	49 ± 6	54 ± 2	54 ± 4
CREAT (mg/dL)	0.42 ± 0.16	0.34 ± 0.22	0.43 ± 0.13	0.35 ± 0.26	0.45 ± 0.10	0.35 ± 0.19	0.46 ± 0.11	0.42 ± 0.09
UA (mg/dL)	2.40 ± 0.52	2.64 ± 0.72	2.24 ± 0.46	2.16 ± 0.28	2.31 ± 0.38	2.23 ± 0.18	2.53 ± 0.41	2.37 ± 0.19
CHOL (mg/dL)	89 ± 3	91 ± 2	87 ± 3	86 ± 4	88 ± 4	87 ± 4	92 ± 2	88 ± 5
PROT (g/dL)	7.03 ± 0.18	6.84 ± 0.41	7.03 ± 0.11	6.58 ± 0.44	6.48 ± 0.78	6.21 ± 0.98	6.82 ± 0.53	6.49 ± 0.51

n = number of mice per group, WBC = white blood cell count, N = neutrophils, L = lymphocytes, M = monocytes, E = eosinophils, B = basophils, RBC = red blood cell count, Hb = haemoglobin, PCV = packed cell volume (haematocrit), PLT = platelet count, ASAT = aspartate aminotransferase, ALAT = alanine aminotransferase, CREAT = serum creatinine, UA = uric acid, CHOL = total cholesterol, PROT = total proteins.

**Table 6:** Equilibrium swelling degree (*Q*_esw_) and kinetic parameters of HA/PVA hydrogels in different media.

Sample Name	pH	*Q*_esw_ (%)	*n*_sw_	*k*_sw_ (min)^−n^	*R*^2^
HAPVA3cy	2	1090.36	0.125	0.52	0.997
HAPVA3cy	4	910.90	0.27	2.94	0.96
HAPVA3cy	5.4	1346.50	0.27	4.06	0.99
HAPVA3cy	7.4	778.90	0.22	2.66	0.97
HAPVA3cy-MTX	2	906.73	0.116	0.51	0.995
HAPVA3cy-MTX	4	1230.10	0.21	5.2	0.99
HAPVA3cy-MTX	5.5	1749.90	0.38	4.6	0.99
HAPVA3cy-MTX	7.4	894.10	0.16	4.89	0.98
HAPVA2cy	4	728.70	0.35	2.53	0.93
HAPVA2cy	5.5	1244.10	0.36	2.77	0.97
HAPVA2cy	7.4	435.80	0.26	2.61	0.97
HAPVA2cy-MTX	4	792.90	0.23	3.47	0.97
HAPVA2cy-MTX	5.5	1412.60	0.32	4.1	0.99
HAPVA2cy-MTX	7.4	640.00	0.21	2.93	0.98

**Table 7:** In vitro drug release characteristics, including kinetic parameters (*n*_r_ and *k*_r_) of HA/PVA hydrogels at different pHs.

Samples name	pH	Maximum amount released (%)	*t*_eqr_ (min)	*t*_1/2r_ (min)	*n*_r_	*R*^2^_nr_	*K*_r_ (min)^−n^	*R*^2^_kr_
HAPVA2cy-MTX	2	37.4	390	27	0.073	0.97	0.72	0.99
HAPVA3cy-MTX	2	36.5	180	29	0.097	0.99	0.75	0.99
HAPVA2cy-MTX	3.5	36.3	330	24.4	0.079	0.95	0.68	0.99
HAPVA3cy-MTX	3.5	35.3	240	26.3	0.110	0.97	0.67	0.99
HAPVA2cy-MTX	5.5	48.2	450	35	0.087	0.97	0.59	0.99
HAPVA3cy-MTX	5.5	44.5	250	35	0.098	0.99	0.62	0.99
HAPVA2cy-MTX	7.4	53.4	600	46	0.044	0.99	0.74	0.99
HAPVA3cy-MTX	7.4	51.5	480	44	0.054	0.98	0.78	0.99

*n*_r_—diffusional exponent that characterizes the drug release mechanism; *k*_r_—release kinetic constant; *R*^2^_nr_/*R*^2^_kr_—correlation coefficient; *t*_1/2r_—time for the drug concentration to decrease to one half of its original value; *t*_eqr_—time at the maximum drug released.
